# TRIM16 employs NRF2, ubiquitin system and aggrephagy for safe disposal of stress-induced misfolded proteins

**DOI:** 10.15698/cst2018.12.169

**Published:** 2018-11-16

**Authors:** Kautilya Kumar Jena, Subhash Mehto, Srinivasa Prasad Kolapalli, Parej Nath, Swati Chauhan, Santosh Chauhan

**Affiliations:** 1 Cell Biology and Infectious Diseases Unit, Institute of Life Sciences, Bhubaneswar, 751023, India.; 2 School of Biotechnology, KIIT University, Bhubaneswar, 751023, India.

**Keywords:** TRIM16, NRF2/NFE2L2, p62/SQSTM1, autophagy, protein aggregates, cancer, oxidative stress, protein homeostasis, protein quality control, aggrephagy, proteotoxic stress

## Abstract

The cellular stresses, genetic mutations, and environmental factors can critically affect the protein quality control checkpoints resulting in protein misfolding. Molecular chaperones play a crucial role in maintaining the healthy proteome by refolding the misfolded proteins into the native functional conformations. However, if they fail to refold the misfolded proteins into the native state, they are targeted by proteolytic systems for degradation. If the misfolded protein numbers increase more than what a cell can resolve, they get converted protein aggregates/inclusion bodies. The inclusion bodies are less cytotoxic than misfolded proteins. The enhanced production of misfolded proteins and protein aggregates are linked to several diseases collectively termed proteinopathies, which includes several neurodegenerative disorders. The understanding of molecular mechanisms that regulate the turnover of protein aggregates will pave path for therapeutic interventions of proteinopathies. In a recent report, we showed that a tripartite motif (TRIM) family protein, TRIM16 streamlines the process of protein aggregates turnover by regulating the NRF2-p62 axis and autophagy.

We found that under oxidative or proteotoxic stress conditions, the TRIM16 is required for biogenesis of protein aggregates from misfolded proteins. In our attempt to investigate the mechanism by which TRIM16 regulates biogenesis of protein aggregates, we found that TRIM16 via NRF2 induces a program of genes that is required for conversion of stress-induced misfolded proteins into protein aggregates. We found that TRIM16 knock out HeLa cells showed reduced expression of NRF2 and p62/SQSTM1 both in basal and stress conditions. We found that TRIM16 interacts with both NRF2 (nuclear factor erythroid 2 like 2) and p62/SQSTM1 and increases their protein stability (**Figure 1**). TRIM16 exploits multiple mechanisms to enhance NRF2 stability and activation. KEAP1 (Kelch-like ECH-associated protein 1) is known to mediate proteasomal degradation of NRF2. Previous studies showed that p62 displaces KEAP1 from NRF2 and also mediates autophagic degradation of KEAP1 and hence positively regulates NRF2 activation. We found that TRIM16 increases p62-NRF2 interaction and displaces KEAP1 from NRF2, thereby releasing NRF2 from the inhibitory complex. TRIM16 also increases KEAP1-p62 interaction, possibly to enhance p62-mediated autophagic degradation of KEAP1. Besides these indirect approaches, TRIM16 directly interacts with NRF2 and induces its K63-linked ubiquitination, which we found is important for stabilizing the NRF2 levels. The activated NRF2, which is a master regulator of the anti-oxidative stress response, induces a program of genes that includes p62, TRIM16 and ubiquitin pathway genes for conversion of oxidative/proteotoxic stress-induced misfolded proteins into protein aggregates (**Figure 1**). In agreement with this, we found that the biogenesis of stress-induced protein aggregates requires NRF2, p62 and ubiquitin pathway genes.

**Figure 1:**
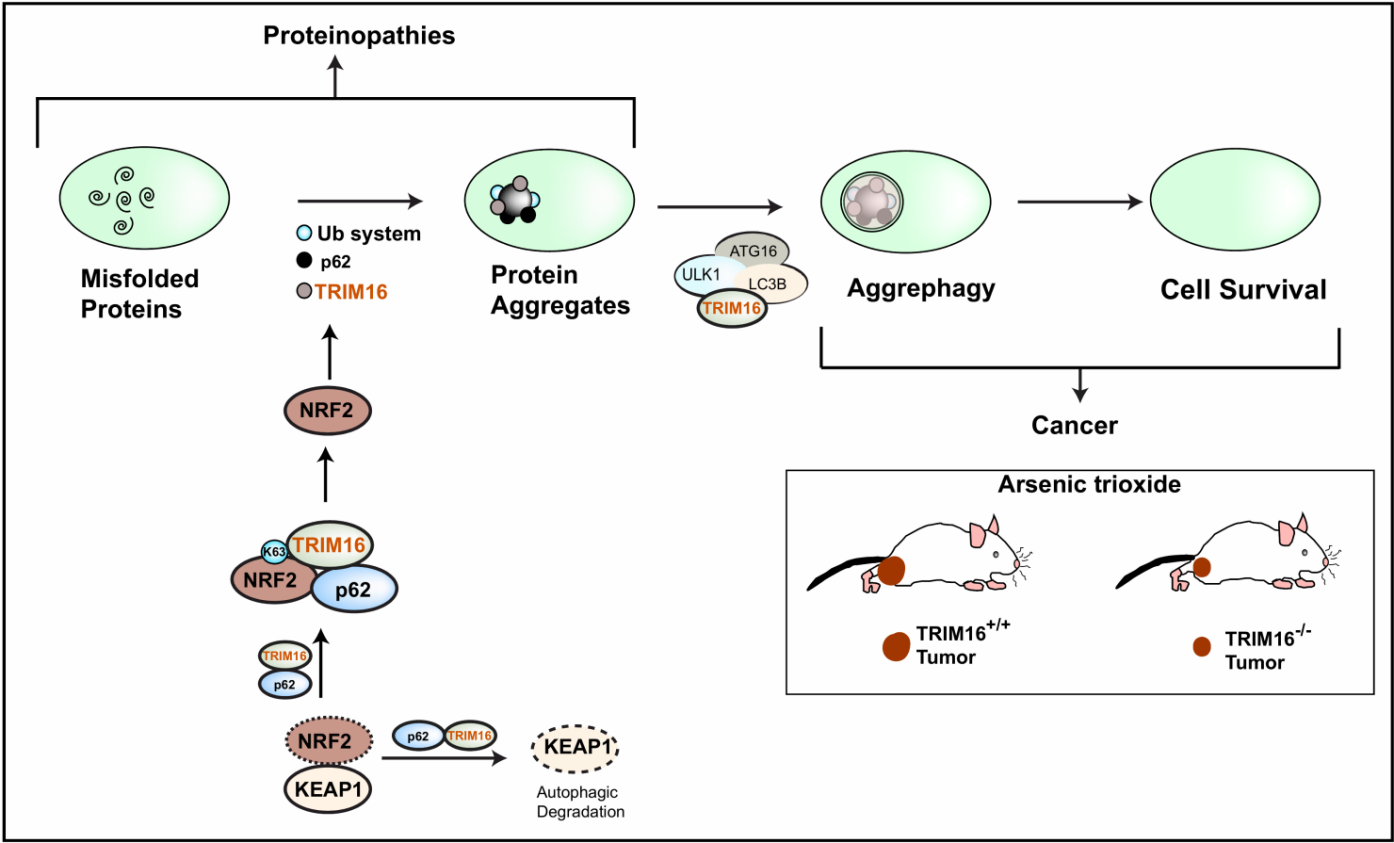
FIGURE 1: TRIM16 maintains protein homeostasis by regulating NRF2 and aggrephagy. The TRIM16 (tripartite motif-containing protein 16) interacts with NRF2 (nuclear factor erythroid 2 like 2) and p62/SQSTM1. TRIM16 displaces KEAP1 (Kelch-like ECH-associated protein 1) and enhances K63-linked ubiquitination of NRF2 to stabilize its expression. The activated NRF2 induces expression of p62, TRIM16 and ubiquitin pathway genes. All the three play an important role in the conversion of stress-induced misfolded proteins into protein aggregates. TRIM16 is present over the protein aggregates and acts as a scaffold protein to bring the autophagy adaptor proteinp62, the autophagy initiation protein ULK1 (Unc-51 like autophagy activating kinase 1), the autophagy elongation protein LC3B (microtubule associated protein 1 light chain 3 beta), and ATG16L1 (Autophagy related 16 like 1) over the protein aggregates for aggrephagy. This mechanism of turnover of misfolded proteins could be protective in proteinopathies. However, cancer cells exploit this mechanism for their survival. Therefore, we found that in a xenograft mouse model, TRIM16 knockout (TRIM16^-/-^) tumors were regressed after exposure to oxidative stress (Arsenic trioxide).

Selective degradation of protein aggregates by autophagy is called aggrephagy. Previous studies demonstrated the roles of TRIM family proteins in a different kind of selective autophagy. TRIM16 previously was shown to interact with Galectin-3 and to assemble the autophagy machinery at the site of endomembrane damages. In this study, we found that TRIM16 is present over the stress-induced protein aggregates where it acts as a scaffold protein to recruit ubiquitin, p62, and LC3B (microtubule-associated protein 1 light chain 3 beta). TRIM16 complexes with ULK1 (Unc-51 like autophagy activating kinase 1) and ATG16L1 (Autophagy-related 16 like 1) over the protein aggregates and thus may help in *de novo* autophagosome biogenesis, resulting in sequestration of protein aggregates and aggrephagy (**Figure 1**). Thus, TRIM16 uses a two-pronged approach to safely dispose of oxidative/proteotoxic stress-induced cytotoxic misfolded proteins. First, it activates a NRF2-mediated program of genes that converts misfolded proteins into less toxic protein aggregates. Second, these protein aggregates are degraded by TRIM16-mediated aggrephagy. By streamlining this process, TRIM16 maintains proteostasis and protects cells from oxidative and proteotoxic stress-induced cell death (**Figure 1**).

This study could have potential implications in proteinopathies and aging. The NRF2 is well recognized for its protective role in neurodegeneration and was found to be strongly inhibited in neurodegenerative diseases. The reactivation of NRF2 is considered as one important therapeutic approach for neurodegenerative diseases. A large number of studies showed that autophagy plays a crucial role in the degradation of protein aggregates and dysfunctional autophagy contributes to the pathology of several neurodegenerative disorders. TRIM16 can regulate both NRF2 and autophagy and protect the cells under stress conditions. Thus, TRIM16 could be a potential therapeutic target for proteinopathies and ageing.

Due to their high metablic rates, cancer cells are constantly under oxidative and proteotoxic stress conditions. This perturbs the protein quality control and increases the number of misfolded proteins and protein aggregates in cancer cells. For this reason, the autophagy and proteasome system is induced in a majority of cancer types. The inhibition of both of the systems is currently considered as one of the important approaches to kill cancer cells. A very recent study found that enhanced capacity to degrade misfolded proteins alleviates the oxidative stress associated with oncogenic growth and is required for both the initiation and maintenance of malignancy. They also found that NRF2 and TRIMs, especially TRIM11, play an important role in the enhanced proteasomal degradation of misfolded proteins. In our study, we found that TRIM16 utilizing p62-NRF2 axis and autophagy program executes a complete turnover of misfolded proteins. The tumors formed by TRIM16 knockout HeLa cells were of the same size as of wild-type cells. However, the exposure of oxidative stress rapidly reduced the size of TRIM16 knockout tumors without affecting the wild-type tumors (**Figure 1**). These data suggest that due to the crippled proteolytic system in the TRIM16 knock out cells, they were not able to handle oxidative stress and died. The enhanced clearance of oxidative stress-induced misfolded proteins by TRIM16 helps cancer cells to survive in a xenograft tumor mouse model. We conclude that incapacitating the proteolytic machinery by inhibiting NRF2 and autophagy (or TRIM16) could be a novel therapeutic approach against cancer.

